# Analysis of risk factors for differentiation syndrome in patients with acute promyelocytic leukemia

**DOI:** 10.1097/MD.0000000000046458

**Published:** 2025-12-12

**Authors:** Mingwan Zhang, Huiqing He, Yongbin Ye, Ziwen Guo

**Affiliations:** a Department of Hematology, Zhongshan City People’s Hospital, Zhongshan, Guangdong Province, China.

**Keywords:** acute promyelocytic leukemia, all-trans retinoic acid, arsenic trioxide, differentiation syndrome, multivariate logistic regression, risk factors

## Abstract

Differentiation syndrome (DS) is a potentially life-threatening complication during induction therapy for acute promyelocytic leukemia (APL). Despite therapeutic advances, early identification of patients at high risk for DS remains clinically critical. This study aimed to investigate clinical, laboratory, and treatment-related risk factors associated with DS in patients with newly diagnosed APL. This retrospective observational study included 316 adult patients diagnosed with APL between January 2014 and December 2024. Diagnosis was confirmed by cytomorphology, immunophenotyping, cytogenetics, and detection of the PML-RARA fusion gene. All patients received induction therapy with all-trans retinoic acid, arsenic trioxide, or both. Clinical and laboratory parameters were compared between DS (n = 96) and non-DS (n = 220) groups. Logistic regression analysis was used to identify independent predictors of DS. The incidence of DS was 30.4%. Compared to the non-DS group, DS patients exhibited significantly higher white blood cell (WBC) counts, neutrophil counts, and percentages of bone marrow and peripheral blood blasts (all *P* < .001). Coagulation abnormalities (prolonged prothrombin time, reduced fibrinogen) and hypoalbuminemia were more frequent in the DS group. Multivariate analysis identified 3 independent risk factors: absence of prophylactic corticosteroid use (odds ratio [OR] 0.10, 95% confidence interval [CI] 0.03–0.35), elevated peak WBC during induction (OR 1.07, 95% CI 1.03–1.11), and lower baseline serum albumin (OR 0.86, 95% CI 0.79–0.93). Elevated peak WBC, hypoalbuminemia, and lack of prophylactic corticosteroids are independent predictors of DS in APL patients. These findings underscore the importance of early risk stratification and preventive strategies to mitigate DS risk during induction therapy.

## 1. Introduction

Acute promyelocytic leukemia (APL), a distinct subtype of acute myeloid leukemia (AML), is characterized by the reciprocal translocation t(15;17)(q24;q21), resulting in the PML-RARA fusion gene. This molecular hallmark disrupts normal myeloid differentiation and underpins the pathogenesis of APL. The introduction of all-trans retinoic acid (ATRA) and arsenic trioxide (ATO) has revolutionized the treatment landscape of APL, enabling high rates of complete remission and long-term survival.^[1,2]^ However, the success of differentiation therapy is often compromised by a potentially life-threatening complication known as differentiation syndrome (DS), previously referred to as retinoic acid syndrome.a potentially life-threatening condition marked by fever, dyspnea, pulmonary infiltrates, hypotension, and organ dysfunction.^[3–5]^

The incidence of DS among APL patients undergoing induction therapy ranges from 15% to 25%, although variability exists depending on the diagnostic criteria and treatment regimens used. Despite its clinical significance, the precise risk factors associated with the development of DS remain incompletely defined. Prior studies have identified potential predictors including elevated baseline leukocyte counts, renal dysfunction, body mass index, microgranular morphology, and the presence of FLT3-ITD mutations.^[6,7]^ However, results have been inconsistent, and predictive models lack external validation. Furthermore, emerging evidence suggests that inflammatory biomarkers, immunophenotypic profiles, and genetic polymorphisms may also contribute to individual susceptibility, yet these factors have not been systematically integrated into risk stratification approaches. Understanding the predisposing factors for DS is critical for early identification of high-risk patients and timely prophylactic intervention.^[8,9]^ Current clinical guidelines recommend empirical administration of corticosteroids at the first signs of DS; however, the absence of reliable predictive indicators hampers the implementation of risk-adapted management strategies. A more refined understanding of DS pathogenesis and its associated risk factors could inform the development of personalized therapeutic approaches and improve patient outcomes.^[10,11]^

Therefore, this study aimed to systematically analyze risk factors for DS in a large cohort of newly diagnosed APL patients, with particular focus on clarifying whether baseline serum albumin represents a novel and clinically meaningful predictor. By addressing this gap, our findings may refine risk stratification and inform preventive strategies in the management of APL.

## 2. Methods

### 2.1. Study design

This research was approved by the Ethics Committee of Zhongshan City People’s Hospital. This retrospective observational study was conducted to evaluate risk factors associated with DS in patients diagnosed with APL between January 2014 and December 2024. Patients were eligible for inclusion if they met the following criteria: confirmed diagnosis of APL based on cytomorphological evaluation, immunophenotyping, cytogenetic analysis, and molecular detection of the t(15;17)(q24;q21) translocation or the PML-RARA fusion gene, in accordance with the World Health Organization (WHO) classification of hematopoietic and lymphoid neoplasms; receipt of induction therapy containing ATRA, ATO, or a combination of both agents as part of standard frontline treatment; age ≥ 18 years at the time of diagnosis; and availability of complete clinical and laboratory data necessary for analysis. Exclusion criteria comprised the presence of therapy-related or secondary APL arising from prior hematologic malignancies or exposure to cytotoxic treatments, the coexistence of other hematologic or solid malignancies, and severe baseline hepatic or renal dysfunction (defined as liver transaminase levels ≥ 5 times the upper limit of normal or creatinine clearance < 30 mL/min). Additionally, patients who died within 48 hours of initiating induction therapy due to causes unrelated to DS were excluded to avoid misclassification. A total of 316 patients were included in the final analysis. Based on the occurrence of differentiation syndrome during induction therapy, patients were categorized into the DS group (n = 96) and the non-DS group (n = 220). Informed consent was obtained from all participants prior to data collection, and the study protocol was approved by the institutional ethics committee in accordance with the Declaration of Helsinki. This was a single-center retrospective study conducted at Zhongshan City People’s Hospital, which may limit the generalizability of our findings to other settings.

Given the retrospective design, the sample comprised all consecutive eligible patients within the study window. With 316 patients and 96 differentiation syndrome (DS) events (~30%), the events-per-variable (EPV) criterion (EPV ≥ 10) supports inclusion of ≥ 9 parameters in multivariable logistic regression. Our final model retained 3 independent predictors, satisfying these recommendations. A post hoc power assessment further suggests that, under plausible correlations among covariates and α = 0.05, this sample provides > 80% power to detect clinically meaningful odds ratios for key continuous predictors. Collectively, these considerations indicate adequate power for the primary analyses

### 2.2. Diagnostic criteria for differentiation syndrome

The diagnosis of DS in patients with APL was based on clinical criteria proposed by Frankel et al, which remain widely accepted in both clinical trials and retrospective analyses.^[12]^ A diagnosis of DS was established when a patient developed 3 or more of the following clinical manifestations during induction therapy with ATRA and/or ATO:

1. Unexplained fever (≥38°C)
2. Respiratory distress, including dyspnea or hypoxia
3. Pulmonary infiltrates or pleural effusion observed on imaging
4. Unexplained weight gain (>5 kg)
5. Unexplained hypotension
6. Acute renal dysfunction
7. Peripheral edema

### 2.3. Induction and supportive treatment strategies based on risk stratification

For patients with low- and intermediate-risk APL, the standard induction therapy consisted of ATRA combined with ATO. Specifically, ATRA was administered orally at a dose of 25 mg/m²/day, and ATO was administered intravenously at 0.16 mg/kg/day. Treatment was continued until morphological complete remission (CR) was achieved. For high-risk patients—defined by a presenting white blood cell (WBC) count > 10 × 10⁹/L—anthracycline-based chemotherapy (e.g., idarubicin or daunorubicin) was added to the ATRA + ATO backbone during induction. Following achievement of CR, these patients continued to receive consolidation chemotherapy incorporating anthracyclines to reduce relapse risk and improve long-term disease control. During induction therapy, if a diagnosis of DS was established, based on clinical and radiological criteria, prompt intervention was initiated. The management protocol included immediate reduction or temporary discontinuation of ATRA, close monitoring of fluid balance, and respiratory function assessment. Intravenous dexamethasone was administered at a dose of 10 mg twice daily as first-line therapy for DS, in combination with supportive care measures such as oxygen supplementation, diuretics, and intensive monitoring.

### 2.4. Data collection and clinical parameters

Baseline data were collected for all patients, including comorbidities, Eastern Cooperative Oncology Group (ECOG) performance status, and body mass index (BMI). Laboratory parameters at diagnosis included WBC, absolute neutrophil count (ANC), hemoglobin (HGB), platelet count (PLT), activated partial thromboplastin time (APTT), prothrombin time (PT), fibrinogen (FIB), D-dimer, lactate dehydrogenase (LDH), serum creatinine (Cr), and albumin (ALB). Disease-related variables included the percentage of blasts in bone marrow and peripheral blood, PML-RARA fusion gene subtype, common gene mutations (e.g., FLT3-ITD), immunophenotypic markers, and cytogenetic karyotype. Treatment-related data included the initiation date of ATRA and ATO, type of anthracycline used, peak WBC during induction, occurrence and symptoms of differentiation syndrome (DS), and whether prophylactic corticosteroids were administered.

### 2.5. Statistical analysis

All statistical analyses were performed using SPSS version 25.0 (IBM Corp., Armonk). Continuous variables were summarized as medians and ranges, and between-group comparisons were conducted using the Mann–Whitney *U* test (non-parametric rank-sum test). Categorical variables were expressed as frequencies and percentages, and were compared using the chi-square test or Fisher’s exact test, as appropriate. Univariate and multivariate logistic regression analyses were conducted to identify potential risk factors associated with the development of DS in newly diagnosed APL patients. Variables with a *P*-value < .05 in univariate analysis were entered into the multivariate model. A 2-sided *P*-value < .05 was considered statistically significant.

## 3. Results

### 3.1. Baseline demographic, clinical, and laboratory characteristics

A total of 316 APL patients were included, of whom 96 (30.4%) developed DS during induction. There were no significant differences between the DS and non-DS groups in terms of age, sex distribution, BMI, or ECOG performance status (all *P* > .05). Comorbidities, including hypertension, diabetes, and CHD, were similarly balanced between groups (*P* > .4). However, risk stratification differed markedly: the high-risk category was overrepresented among DS patients (44.8% vs 15.5%; *P* < .001). Laboratory parameters at diagnosis revealed significantly higher median WBC and NEUT counts in the DS cohort (7.45 vs 2.59 × 10⁹/L and 2.02 vs 0.78 × 10⁹/L, respectively; both *P* < .001). Coagulation disturbances were more pronounced in DS patients, with longer PT (19.5 vs 18.3 seconds; *P* = .008) and lower fibrinogen levels (1.19 vs 1.57 g/L; *P* < .001). Hypoalbuminemia was also more frequent in the DS group (median ALB 39.5 vs 40.5 g/L; *P* < .001). Indicators of leukemic burden, including bone marrow and peripheral blast percentages, were significantly elevated in DS patients (median BM blasts 88% vs 85%, *P* < .001; PB blasts 73% vs 52%, *P* = .004). No significant differences were observed in HGB, PLT, APTT, D-dimer, LDH, or Cr. Cytogenetic and molecular features (t[15;17], PML-RARα subtype, FLT3-ITD, WT1 copy number, CD2/CD56 expression) did not differ between groups (Table 1).

**Table 1:** Comparison of baseline characteristics, laboratory parameters, and treatment features in APL patients with and without differentiation syndrome.

Items	Non-DS (n = 220)	DS (n = 96)	*P*-value
Demographics and performance			
Age (yr)	44 (12–75)	46 (12–78)	.450
Sex (male/female)	113/107	63/33	.063
BMI (kg/m²)	24.4 (15.8–35.6)	24.3 (18.9–36.3)	.860
ECOG score 0–1/2–4	210/10	88/8	.089
Risk stratification and comorbidities			
Risk group: Low/Intermediate/High	62/124/34	16/37/43	<.001
Hypertension (no/yes)	204/16	89/7	.480
Diabetes (no/yes)	203/17	92/4	.930
CHD (no/yes)	214/6	93/3	.680
Hematologic and biochemical data			
WBC (×10^9^/L)	2.59 (0.32–89.2)	7.45 (0.40–200.0)	<.001
NEUT (×10^9^/L)	0.78 (0.00–92.3)	2.02 (0.82–194.0)	<.001
HGB (g/L)	80 (46–134)	84 (46–128)	.970
PLT (×10¹²/L)	28 (5–150)	47 (10–84)	.330
PT (s)	18.3 (10.0–41.0)	19.5 (11.0–74.1)	.008
APTT (s)	28.5 (19.8–38.8)	30.0 (14.0–42.5)	.530
FIB (g/L)	1.57 (0.15–17.5)	1.19 (0.30–5.64)	<.001
D-dimer (mg/L)	7.75 (0.10–165.0)	11.10 (0.12–145.0)	.070
LDH (U/L)	295 (38–4 800)	310 (36–6 500)	.150
Cr (μmol/L)	61.1 (30–176)	65 (26–515)	.050
ALB (g/L)	40.5 (23.0–58.0)	39.5 (24.0–50.0)	<.001
BM blasts (%)	85 (21–98)	88 (37–99)	<.001
PB blasts (%)	52 (1–100)	73 (0.2–100)	.004
Cytogenetics and molecular			
t(15;17) (yes/no)	212/8	90/6	.084
PML-RARα subtype (L/S/V)	80/44/3	68/33/2	.908
FLT3-ITD (yes/no)	4/216	2/94	.926
WT1 copies (high/medium-low)	42/178	15/81	.570
CD2 (pos/neg)	5/215	4/92	.144
CD56 (pos/neg)	30/190	8/88	.518
Treatment details			
ATRA initiation to CR (days)	1 (1–23)	2 (1–32)	.002
ATO initiation to CR (days)	1 (1–16)	1 (1–18)	.270
Anthracycline: Ida/Dauno/None	111/67/42	49/29/18	.606
Prophylactic corticosteroids (yes/no)	78/142	11/85	<.001
DIC at presentation (yes/no)	99/121	66/30	<.001
Peak WBC post-chemo (×10^9^/L)	13.0 (1.04–65.0)	30.2 (4.13–200.0)	<.001

APTT = activated partial thromboplastin time, ATO = arsenic trioxide, ATRA = all-trans retinoic acid, BM = bone marrow, BMI = body mass index, CHD = coronary heart disease, Cr = creatinine, DIC = disseminated intravascular coagulation, DS = differentiation syndrome, ECOG = Eastern Cooperative Oncology Group, FIB = fibrinogen, HGB = hemoglobin, Ida = idarubicin, LDH = lactate dehydrogenase, PB = peripheral blood, PLT = platelet, PT = prothrombin time, S = short PML-RARα subtype, V = variable PML-RARα subtype, WBC = white blood cell, WT1 = Wilms’ tumor-1 gene copy number.

### 3.2. Treatment-related features

Time to CR following ATRA initiation was modestly prolonged in DS patients (median 2 vs 1 day; *P* = .002). This delay may be attributable to treatment interruptions or supportive interventions required during DS episodes, whereas ATO timing did not differ. Although the overall use of anthracyclines (idarubicin or daunorubicin) was comparable, DS patients were significantly less likely to receive prophylactic corticosteroids (11.5% vs 35.5%; *P* < .001) and more likely to present with DIC (68.8% vs 45.0%; *P* < .001). Finally, the peak WBC count following induction was substantially higher in the DS cohort (30.2 vs 13.0 × 10⁹/L; *P* < .001) (Table 1).

### 3.3. Univariate analysis of risk factors for differentiation syndrome

8 In univariate logistic regression, high-risk stratification emerged as a strong predictor of DS, with patients in the high-risk group demonstrating a 5-fold increase in odds compared with the low-risk group (odds ratio [OR] 5.24, 95% confidence interval [CI] 2.42–10.66; *P* < .001), whereas intermediate-risk status showed no significant difference (*P* = .580). Treatment-related factors, including each additional day’s delay to ATRA initiation, conferred a modest increase in DS risk (OR 1.12 per day, 95% CI 1.04–1.20; *P* = .002). Coagulopathy at presentation (DIC) more than doubled the odds of DS (OR 2.44, 95% CI 1.49–4.00; *P* < .001). Elevated leukocyte parameters, namely WBC (OR 1.04 per 10⁹/L, 95% CI 1.02–1.06; *P* < .001) and NEUT counts (OR 1.08, 95% CI 1.03–1.11; *P* < .001) at diagnosis, as well as peak WBC after chemotherapy (OR 1.06 per 10⁹/L, 95% CI 1.04–1.08; *P* < .001), were all significantly associated with DS. Among coagulation and biochemical variables, prolonged PT (OR 1.08 per second, 95% CI 1.02–1.18; *P* = .008), reduced fibrinogen (OR 0.69, 95% CI 0.53–0.88; *P* = .007), and lower albumin (OR 0.93 per g/L, 95% CI 0.88–0.98; *P* < .001) predicted DS occurrence. Finally, higher proportions of bone marrow and peripheral blasts also increased DS risk (BM blasts: OR 1.04 per %, 95% CI 1.01–1.07; *P* < .001; PB blasts: OR 1.02, 95% CI 1.00–1.02; *P* = .008) (Table 2).

**Table 2:** Risk factors for differentiation syndrome in APL patients: univariate and multivariate logistic regression.

Items	Univariate OR (95% CI)	*P*-value	Multivariate OR (95% CI)	*P*-value
Risk group				
Intermediate vs Low	1.18 (0.59–2.41)	.580	—	—
High vs Low	5.24 (2.42–10.66)	<.001	—	—
Treatment				
ATRA initiation interval (days)	1.12 (1.04–1.20)	.002	—	—
Prophylactic hormone use (yes vs no)	0.27 (0.14–0.51)	<.001	0.10 (0.03–0.35)	<.001
Coagulopathy				
DIC at presentation (yes vs no)	2.44 (1.49–4.00)	<.001	—	—
Hematologic parameters				
WBC at diagnosis (×10^9^/L)	1.04 (1.02–1.06)	<.001	—	—
NEUT at diagnosis (×10^9^/L)	1.08 (1.03–1.11)	<.001	—	—
Peak WBC after chemotherapy (×10^9^/L)	1.06 (1.04–1.08)	<.001	1.07 (1.03–1.11)	<.001
Coagulation and biochemistry				
PT (s)	1.08 (1.02–1.18)	.008	—	—
FIB (g/L)	0.69 (0.53–0.88)	.007	—	—
ALB (g/L)	0.93 (0.88–0.98)	<.001	0.86 (0.79–0.93)	.007
Blast proportions				
BM blasts (%)	1.04 (1.01–1.07)	<.001	—	—
PB blasts (%)	1.02 (1.00–1.02)	.008	—	—

ALB = albumin, ATRA = all-trans retinoic acid, BM = bone marrow, CI = confidence interval, DIC = disseminated intravascular coagulation, DS = differentiation syndrome, FIB = fibrinogen, NEUT = neutrophil count, OR = odds ratio, PB = peripheral blood, PT = prothrombin time, WBC = white blood cell.

### 3.4. Multivariate analysis of independent predictors

When variables significant in univariate analysis were entered into a multivariate logistic regression model, 3 factors remained independently associated with DS. Prophylactic corticosteroid use conferred a marked protective effect, reducing DS odds by approximately 90% (OR 0.10, 95% CI 0.03–0.35; *P* < .001). Peak WBC following induction therapy persisted as a significant risk factor (OR 1.07 per 10⁹/L, 95% CI 1.03–1.11; *P* < .001), underscoring the role of leukocyte burden in DS pathogenesis. Additionally, hypoalbuminemia was independently predictive of DS (OR 0.86 per g/L, 95% CI 0.79–0.93; *P* = .007), highlighting the importance of baseline nutritional and protein status (Table 2).

## 4. Discussion

This study analyzed the risk factors for DS during induction therapy in 316 patients with APL, and the findings have important clinical implications. We observed an overall DS incidence of approximately 30% (96/316), which is consistent with previously reported rates in large-scale studies (approximately 25%). Patients who developed DS exhibited significantly elevated baseline WBC counts and absolute neutrophil counts, indicating that a high leukocyte burden at diagnosis is a major risk factor for DS development. This observation aligns with the well-established conclusion in the literature that elevated baseline WBC is one of the strongest predictors of DS in APL patients. For instance, a study by Montesinos et al involving 739 APL patients demonstrated that WBC > 5 × 10⁹/L at diagnosis significantly increased the risk of developing severe DS in multivariate analysis.^[13]^

A high leukemic burden implies a substantial pool of promyelocytes within the body. Upon exposure to differentiation agents such as ATRA, a large number of these cells undergo synchronous differentiation and secrete pro-inflammatory cytokines. This cytokine release can compromise endothelial integrity and increase vascular permeability, triggering a systemic inflammatory response that contributes to DS pathogenesis. This mechanism explains why we observed a higher proportion of blasts in both bone marrow and peripheral blood among patients who developed DS.^[14,15]^ While several prior studies have reported associations between increased peripheral blasts and DS incidence, an observation also supported by our univariate analysis, these parameters did not retain significance in multivariate models, likely due to collinearity with total WBC count. Another key finding of this study is the independent association between hypoalbuminemia and DS development. Baseline serum albumin levels were significantly lower in the DS group, and multivariate analysis showed that each 1 g/L reduction in albumin was associated with a significantly increased risk of DS (OR 0.86, *P* = .007). Hypoalbuminemia may reflect poor nutritional status or a heightened inflammatory state, both of which can compromise vascular oncotic pressure, increase interstitial fluid accumulation, and exacerbate DS-related manifestations such as edema and hypotension. This finding is in line with earlier studies. For example, De la Serna et al identified low albumin as an independent predictor of DS-related mortality during APL induction therapy.^[16]^

It is noteworthy that the ECOG performance status in our cohort did not significantly differ between the DS and non-DS groups. In contrast, prior studies have suggested that ECOG > 1 is associated with a higher risk of DS-related complications. The discrepancy may be attributed to the overall favorable performance status of our study population, where few patients had ECOG scores greater than 1, thereby limiting the detection of its prognostic value in our cohort. In addition, our results revealed more pronounced coagulation abnormalities among DS patients at diagnosis.^[17,18]^ Specifically, patients with DS had significantly prolonged prothrombin time (PT), lower fibrinogen levels, and a higher incidence of disseminated intravascular coagulation (DIC) (68.8% vs 45.0%). These findings suggest a possible relationship between coagulopathy and DS occurrence. It is plausible that the biologic characteristics of APL blasts, which disrupt hemostasis and promote fibrinolysis, may exacerbate systemic inflammation and endothelial dysfunction, thereby facilitating the development of DS. Although previous studies have not identified coagulation parameters as independent predictors of DS, our data highlight the potential value of baseline coagulation profiling as an adjunct for DS risk stratification. Further prospective validation is warranted.^[19,20]^

From a therapeutic perspective, we observed that patients who did not receive prophylactic corticosteroids were significantly more likely to develop DS. Only 11.5% of DS patients received prophylactic corticosteroids during induction, compared to 35.5% in the non-DS group. Multivariate analysis confirmed this protective association, with corticosteroid prophylaxis reducing the odds of DS by approximately 90% (OR 0.10, *P* < .001). This observation is supported by clinical experience and prior research. In the aforementioned study by Montesinos et al., systematic prophylactic use of prednisone was associated with a reduced incidence of severe DS. Current treatment guidelines also recommend prophylactic corticosteroids for high-risk APL patients, particularly those with elevated baseline WBC or receiving ATRA-ATO combination therapy. The protective mechanism of corticosteroids likely involves early suppression of the inflammatory cytokine cascade and stabilization of capillary permeability, thereby preventing the onset or progression of DS. Our findings strongly reinforce the clinical utility of this intervention.^[21,22]^

It is important to emphasize that our study did not identify any significant associations between cytogenetic or molecular abnormalities and DS occurrence. Specifically, classical t(15;17) translocation status, PML-RARα isoforms, FLT3-ITD mutation, and CD2/CD56 expression did not differ between DS and non-DS groups. These results corroborate previous reports suggesting that, apart from leukocyte burden, there are no universally accepted molecular biomarkers for predicting DS risk in APL. While some small-scale studies have suggested potential roles for immunophenotypic markers such as CD56 positivity or certain genetic alterations, the overall level of evidence remains insufficient. Our findings underscore the continued relevance of conventional clinical markers, such as WBC count and albumin level, in risk stratification for DS. Further studies integrating genomic, proteomic, and inflammatory profiling are needed to advance biomarker discovery in this field.

Our findings are consistent with other studies, while also extending previous work by identifying baseline hypoalbuminemia as an independent predictor of DS in an East Asian population. This observation complements recent evidence from Cicconi et al., who highlighted leukocytosis as a predictor, thereby underscoring the novelty of our integrated clinical risk model.

Our study highlights several clinically actionable risk factors that can aid in early identification and management of patients at risk for DS. Specifically, patients presenting with elevated WBC (>10 × 10⁹/L), high marrow or peripheral blast percentages, coagulation abnormalities, and low albumin levels should be considered high-risk for DS. These patients may benefit from closer monitoring during induction therapy, including frequent assessment of respiratory status, fluid balance, and early signs of DS (e.g., fever, edema, pulmonary infiltrates). Prophylactic corticosteroid administration, particularly in patients with high leukemic burden, should be strongly considered to reduce the incidence and severity of DS.

This study has several strengths. The relatively large sample size (n = 316) enabled reliable multivariate analysis with adequate statistical power. Utilizing a single-center cohort ensured consistency in diagnostic standards, therapeutic strategies, and data integrity. The comprehensive evaluation of clinical, hematological, and treatment-related variables allowed for the identification of key independent predictors of DS, including lack of prophylactic corticosteroid use, elevated peak WBC following induction, and low serum albumin levels. Notably, this study adds novel evidence by highlighting hypoalbuminemia as an independent risk factor for DS in an East Asian APL population.

However. This study has several limitations. First, as a retrospective single-center analysis, it is susceptible to selection bias and unmeasured confounding factors, which may influence the observed associations. Second, differentiation syndrome was evaluated as a binary outcome; the lack of stratification by severity (mild vs severe DS) may have masked differential predictors for distinct clinical phenotypes. Third, while the relatively large sample size strengthens statistical power, the findings may not be fully generalizable beyond our institution. Future research should include prospective, multi-center cohorts to validate these findings and minimize selection bias. Additionally, integration of molecular and immunophenotypic markers into predictive models may enhance risk stratification for DS and guide individualized preventive strategies.

## 5. Conclusions

In conclusion, elevated peak WBC, hypoalbuminemia, and lack of prophylactic corticosteroid use were identified as independent predictors of DS in APL patients. Early recognition of these risk factors and timely preventive interventions, particularly corticosteroid prophylaxis, are essential to reduce DS incidence and ensure optimal treatment outcomes.

## Author contributions

**Conceptualization:** Mingwan Zhang, Huiqing He, Yongbin Ye, Ziwen Guo.

**Data curation:** Mingwan Zhang, Ziwen Guo.

**Formal analysis:** Mingwan Zhang.

**Investigation:** Mingwan Zhang, Huiqing He, Ziwen Guo.

**Methodology:** Mingwan Zhang, Huiqing He, Yongbin Ye.

**Supervision:** Huiqing He, Ziwen Guo.

**Validation:** Huiqing He, Yongbin Ye, Ziwen Guo.

**Visualization:** Huiqing He, Yongbin Ye, Ziwen Guo.

**Writing – original draft:** Mingwan Zhang, Ziwen Guo.

**Writing – review & editing:** Mingwan Zhang, Ziwen Guo.
